# Extent of Anaemia among Preschool Children in EAG States, India: A Challenge to Policy Makers

**DOI:** 10.1155/2014/868752

**Published:** 2014-07-23

**Authors:** Rakesh Kumar Singh, Shraboni Patra

**Affiliations:** International Institute for Population Sciences, Mumbai 400088, India

## Abstract

*Background.* India is the highest contributor to child anemia. About 89 million children in India are anemic. The study determines the factors that contributed to child anemia and examines the role of the existing programs in reducing the prevalence of child anemia particularly in the EAG states. *Methods.* The data from the latest round of the National Family Health Survey (NFHS-3) is used. Simple bivariate and multinomial logistics regression analyses are used. *Results.* About 70% children are anemic in all the EAG states. The prevalence of severe anemia is the highest (6.7%) in Rajasthan followed by Uttar Pradesh (3.6%) and Madhya Pradesh (3.4%). Children aged 12 to 17 months are significantly seven times (RR = 7.99, *P* < 0.001) more likely to be severely anemic compared to children of 36 to 59 months. Children of severely anemic mothers are also found to be more severely anemic (RR = 15.97, *P* < 0.001) than the children of not anemic mothers. *Conclusions.* The study reveals that the existing government program fails to control anemia among preschool children in the backward states of India. Therefore, there is an urgent need for monitoring of program in regular interval, particularly for EAG states to reduce the prevalence of anemia among preschool children.

## 1. Introduction

Worldwide, anemia among preschool children is one of the serious public health problems. Globally 1.62 billion people are anemic, while among the preschool children the prevalence of anemia is 47.4% [1]. In India, about 89 million children are anemic [1, 2]. Thus, India is the highest contributor to child anemia among the developing countries [2, 3]. According to the latest national representative survey of India, 70% children are anemic in the age group of 6–59 months, including 3% severely anemic, 40% moderately anemic, and 26% mildly anemic (NFHS 3, 2005-06) [4]. Anemia is the most predominant factor for morbidity and child mortality [5–7], and hence, it is a critical health issue for preschool children in India [8, 9].

Anemia is considered as a proxy indicator of iron deficiency [2, 10] because it is defined as an abnormal iron biochemistry with or without anemia [11]. Iron deficiency is caused by the poor iron intake and low iron bioavailability [1, 2]. Some other factors like vitamin A, vitamin B_12_, hookworm, and malaria infection are found associated with anemia among preschool children [2, 10]. Iron deficiency anemia affects the physical and mental development of the human body [1, 12]. For instance, many studies have shown that iron deficiency reduces the learning capacity of the children aged below five years, decreases attentiveness, and causes low intelligence [13]. Thus, anemia leads to decrease of the actual economic productivity of human resources and ultimately impacts on the development of the country [12, 14]. Few studies have shown that preschool children are more vulnerable to the risk of iron deficiency anemia [15–17]. The prevalence of iron deficiency anemia is the highest among preschool children. In this age group (6–59 months) body grows rapidly and requires high-iron-rich and nutritious food that may not be fulfilled by their normal diet. Low economic status, less education, and poor health of mothers due to meager dietary intake are the main causes of anemia [18, 19].

Numerous studies have been carried out on anemia in India since the 1980's. However, we have found very few studies on child anemia particularly focusing on children aged 6–59 months at the national [1] and regional levels [10]. Although nutritional problem is very common in all states, it is more prevalent and severe among the children below five years in the particular states, whose performances are very poor in respect of the other important demographic and socioeconomic indicators. The Government of India (GOI) has named these states as Empowered Action Groups (EAG) states, which consist of Uttarakhand, Uttar Pradesh, Madhya Pradesh, Bihar, Odisha, Jharkhand, Chhattisgarh, and Rajasthan. The EAG states comprise almost 45% of Indian population [20].

A number of studies have been conducted to show an association between the socioeconomic status (SES) and the prevalence of anemia [10]. Among the different socioeconomic factors, women's level of education and exposure to mass media are found to play a key role in determining their own and their children's health status. Moreover, preventive health care is supposed to be more effective in reducing child morbidity in those areas where accessibility of and affordability for curative health care services are much less than the other region [21]. Hence, to increase the awareness on preventive health care practices, it is always important to understand the background characteristics of women and their children. Therefore, it is obligatory to scrutinize the extent of prevalence of anemia among preschool children and its determinants in the EAG states. Although the etiology of anemia is multifactorial, there is an urgent need to determine the factors that contributed to anemia and to examine the role of the existing program in controlling child anemia especially in the less developed areas like EAG states.

## 2. Data and Methods

### 2.1. Ethics Statement

No ethics statement is needed for this work, as the study is based on an anonymous dataset which is available in the public domain and does not contain any identifiable information on the survey participants.

### 2.2. Sample Size

The study used National Family Health Survey (NFHS-3) data, which was conducted in 2005-06 covering 29 states in India [4]. The NFHS-3 has collected information from a nationally representative sample of 109,401 households, 124,385 women of age group 15–49 years, and 74,369 men aged 15–54 years. It provides a cross-sectional survey data on preschool children and their mother's haemoglobin status, body weight, and demographic and socioeconomic characteristics. The study has considered only the preschool children aged 6–59 months in the EAG states of India. The total sample of the preschool children is 16065 in the EAG states.

### 2.3. Methods

#### 2.3.1. Variables

The study had used several variables to comprehend the differences in the prevalence of anemia among the preschool children and the interrelationships among the variables related to children and their mother's health. The variables of the study are briefly described in the following section.

*Dependent Variables. Anemia level*: Preschool children with any anemia (mild, moderate, and severe) are considered for this study. The cut-off level of anemia (haemoglobin or Hb level) among the preschool children is less than 11 g/dL. Anemia variable, for preschool children of age group 6–59 months, is divided into four categories: (a) severe (<7 g/dL), (b) moderate (7.0–9.9 g/dL), (c) mild (10–10.9 g/dL), amd (d) not anemic (>11 g/dL), and is used for multinomial regression analysis.

*Independent Variables.* The study includes a set of independent variables to understand the extent and differentials in the level of anemia among preschool children and their mother's, and its effect on the outcomes. The independent variables are mainly socioeconomic and demographic characteristics of mothers. The socioeconomic characteristics of the children and their mothers include* age groups of the children* (6–11, 12–17, 18–23, 24–35, and 36–59 months),* age groups of mothers* (15–19, 20–29, 30–39, and 40–49 years),* place of residence* (urban, rural),* Mother's level of education* (no education, up to primary level complete, up to secondary level complete, and high school and above),* father's level of education* (no education, up to primary level complete, up to secondary level complete, and high school and above),* household structure* (nuclear family, joint family),* wealth quintile* (poorest, poorer, middle, richer, and richest),* media exposure* (no, yes),* birth order of the child* (1, 2-3, 4-5, and 6+), and* mother's anemia status* (severe, moderate, mild, and not anemic).

#### 2.3.2. Statistical Analyses

The study used bivariate and multivariate techniques to comprehend the level of anemia among preschool children of age group 6–59 months belonging to the EAG states of India. Multivariate technique like multinomial logit regression analysis is applied to examine the effect of socioeconomic and demographic factors on the level of anemia among preschool children and their mothers.

*Multinomial Logit Regression (MLR) Analysis.* Multinomial regression is the most appropriate technique in a situation where the dependent variables are categorical and have more than two categories. The model allows the study to see the effect of a unit change in the predictors or independent variables on the outcome or dependent variable considering the simultaneous effects of several other variables in the form of the relative risk (RR). The multinomial regression model is a generalized form of the logistic regression model. In the present study, the multinomial regression model is used to analyze the effect of some selected socioeconomic and demographic factors on anemia among preschool children in the EAG states of India. The categories of the level of anemia among preschool children are severe (<7 g/dL), moderate (7.0–9.9 g/dL), mild (10–10.9 g/dL), and not anemic (>11 g/dL).

## 3. Results

### 3.1. Prevalence of Anemia among Preschool Children in the EAG States

Table 1 illustrates that the prevalence of severe anemia among preschool children is the highest in Rajasthan (6.7%) followed by Uttar Pradesh (3.6%) and Madhya Pradesh (3.4%), and it is the lowest in Odisha (1.5%). The prevalence of moderate anemia is the highest in Bihar (47.3%) and the lowest in Uttarakhand (30.8%). The prevalence of moderate anemia is almost more than 40% in all EAG states except Odisha and Uttarakhand. About 30% preschool children are moderately anemic in Bihar, Jharkhand, and Odisha, whereas, in the rest of the states, the percentage is above 20. Among the EAG states, 3% children are found to be severely anemic, 41% are moderately anemic, and about 27% are mildly anemic.

### 3.2. Prevalence and Associated Factors of Anemia among Preschool Children by Their Background Characteristics

Table 2 illustrates that the prevalence of severe anemia has been found more among 12–17 month children (5.2%) as compared to 36–59 month children (3.9%), whereas prevalence of moderate anemia has been found above 50% among 6–23 month children. Approximately, 80% preschool children have any anemia in the EAG states. The children of the mothers of age group 15 to 19 years and 40 to 49 years are found to have severe anemia (4.8% and 6.1%, resp.). Children are found to be less severely anemic (1.6%) of those women who have at least high school and above education, as compared to the children of those women who have no education (3.5%). Mother's level of education plays a significant role in determining the level of anemia among children in the EAG states. Children, belonging to the joint family, are less severely anemic than those who live in nuclear families (2.8% and 3.4%, resp.). About 12.9% preschool children have severe anemia as their mothers are found to be severely anemic. For those women who do not have anemia, their children are found to be less severely anemic (1.8%).

Table 3 provides the estimates from the multinomial regression analysis, used to find out the contributing factors to anemia among preschool children in the EAG states. It shows that children of those mothers who have severe anemia are about 16 times (RR = 15.97, *P* < 0.001) more likely to be severely anemic as compared to the children of not anemic mothers. Children belonging to the richest quintile are less likely to be severely anemic as compared to children who belong to poor or middle wealth quintile. Children are more likely to be severely anemic of those mothers who have no education (RR = 1.71, *P* < 0.001) or up to primary education, (RR = 1.61, *P* < 0.001) compared to the children of mothers with higher education. Children belonging to the nuclear family are more likely to be severely anemic than those who live in a joint family. The adolescent mother's children are two times (RR = 1.99, *P* < 0.001) more likely to be moderately anemic as compared to children of older mothers. Education, wealth quintile, and family structure are found to be significant in controlling the level (severe, moderate, and mild) of anemia.

## 4. Discussion

Iron deficiency anemia among preschool children is a major public health problem in Southeast Asia [10, 22]. According to the World Health Organization (WHO), anemia adds to 324,000 deaths and 12,500,000 disability adjusted life years (DALYs) in this region, which is the highest in the world [11, 13, 23]. The present study has reinforced and extended the previous findings that anemia among preschool children is an important public health problem in India. The study found anemia is clearly widespread among preschool children particularly in the EAG states. The prevalence of anemia among preschool children is about 71% in the EAG states, which is much higher than the other less developed South Asian Countries such as Vietnam [24] and Bangladesh [25]. The highest prevalence of anemia among preschool children is found in Bihar (77.9%), followed by Uttar Pradesh (74%), Madhya Pradesh (74%), Chhattisgarh (72.1%), Rajasthan (70.6%), and Jharkhand (70.5%). Contrary to this, Uttarakhand (61.9%) and Odisha (65.5%) have a lower rate of prevalence among the EAG states. Bharati et al., 2013, have shown the state-wise distribution of the prevalence of anemia among preschool children, which also authenticates the findings of the present study [1].

Among all the EAG states, the prevalence of severe anemia among preschool children is found highest in Rajasthan (6.7%). The severe anemia is a very serious problem because recovery from severe anemia is very rare, and there is a high risk of child mortality [26]. The reason of high prevalence of severe anemia in this region could be a lack of dietary energy in their diet and low protein intake by them [27]. Further, a weak economy of the state declines the availability and accessibility of nutrient rich food to the disadvantaged community [26, 27]. The study has also added that mother's anemia status also determines their children's anemia status. Children of severely anemic mothers are found more severely anemic than the children of not anemic mothers.

According to NFHS-3, the total fertility rate (TFR) in the EAG states is much higher (above three children per women in her entire reproductive lifespan) than the other states [4]. In the poor families, additional child is considered as a helping hand for domestic work and later, as the bread earner. Consequently, the higher number of children in the family increases the requirement for childcare, demand for food, and inadequate supply of nutritional diet to all the children which ultimately make the children more vulnerable to the risk of anemia [10, 28, 29]. Similar evidence emerges from the present study, which supports the fact that prevalence of severe anemia among preschool children increases with the increase of their birth order.

GOI has launched many programs to reduce the anemia level among the vulnerable populations by improving their nutritional status. One of the important programs is the National Nutritional Anemia Prophylaxis Program (NNAPP), launched in 1970 [11, 17]. In 1970, about 20% of maternal deaths occurred due to deficiency of iron and folic acid. Through this program, GOI has implemented and distributed IFA tablets to the pregnant and lactating mothers and children by the health centers [30]. Vijayaraghavan and his team had evaluated the NNAPP in 1990. It was evident that health functionaries are not properly oriented towards the program as many of them are not aware of all beneficiaries under the program. The chemical analysis of the tablets indicates that about 30% of the tablet sample was less than expected levels, and none of them had expected levels of folic acid content [31]. Therefore, the program was redesigned as the National Nutritional Anemia Control Program in 1991. The program was designed for the reduction of the incidence of the anemia among the risk population such as pregnant or lactating women, intrauterine device (IUD) consumers, and children aged 12 to 59 months. According to the program, one tablet of 20 mg iron is essential for children aged 1 to 5 years and 100 *μ*g folic acid for 100 days in a year is required by the anemic children [11].

Initially, the program was to be implemented as part of the Reproductive and Child Health (RCH), but now it is the part of the Integrated Child Development Scheme (ICDS) under the Department of Women and Child Development. Government of India has launched ICDS to improve the nutritional status of the preschool children especially from poor or less developed areas. However, the available studies, assessing the ICDS program, have found no significant effects which can control chronic child malnutrition [32]. In 2005, the World Bank found that the services, provided by the local ICDS centers, did not focus on the youngest child (below three years) who should have benefited from the program. In addition, children from the wealthier family participated more in the evaluation compared to the children from poorer or lower caste households. Inadequate worker skills, absence of equipment, and poor monitoring diverted the program from the objective of the ICDS [33]. Some studies have suggested that the IFA tablets given to the children (below three years) were not easily acceptable and recommended the liquid IFA for young children [34]. In 2007, Government of India has modified policy and replaced tablets by liquid IFA. According to the new policy, young children (6–59 months) will get one milliliter of IFA syrup for 100 days in a year that will contain 20 mg elemental iron and 100 *μ*g folic acid [11]. In 2011, zee research groups conducted an interview with child right officer, Chetanalaya (a Delhi based nongovernment organization), who said “The rise in anemic children in Bihar, Uttar Pradesh, and Madhya Pradesh is attributed to the poor health of pregnant and lactating mothers” [35].

## 5. Conclusion

The study reveals that the prevalence of anemia among preschool children is very high among each of the EAG states in India. Special program at the state level is required to control the prevalence of anemia among preschool children in the EAG states. About 45% population of India lives in the EAG states and a reduction in the prevalence of anemia among these states will inevitably decline the prevalence of anemia at the national level. Anemia remains as a serious health problem due to various causes. The present study also helps to find out the factors, such as higher education of mothers and media exposure, playing an important role in controlling the high prevalence of anemia among preschool children in the EAG states. Hence, the intervention targeting only iron and folic supplements, as we have found in the earlier studies, is not adequate to tackle this problem [36]. Now, it is a big challenge to policy makers and programmer to identify specific strategies to reduce anemia in the backward states. Therefore, there is an urgent need to use multiple interventions and new approaches addressing major preventable causes of anemia among the preschool children. Individual state government should take serious measure to improve the quality of services and to provide nutritional education to mothers to improve their children's health status. In addition, proper monitoring and evaluation of the existing programs, such as ICDS, are required to direct the programs towards their success.

## Figures and Tables

**Table 1:** Prevalence of anaemia among preschool children in EAG states in India, NFHS, 2005-06.

States	Anemia status by haemoglobin levels				Sample size
	Severe	Moderate	Mild	Any anaemic	
Uttarakhand	2.3	30.8	28.8	61.9	887
Rajasthan	6.7	40.8	23.2	70.6	1619
Uttar Pradesh	3.6	45.4	25.0	74.0	4788
Bihar	1.6	47.3	29.0	77.9	1995
Jharkhand	2.0	39.5	29.0	70.5	1303
Odisha	1.5	34.5	29.4	65.5	1413
Chhattisgarh	2.1	45.9	24.1	72.1	1298
Madhya Pradesh	3.4	43.6	27.0	74.0	2762

EAG states	2.9	41.0	26.9	70.8	16065

**Table 2:** Percentage distribution of age 6–59 months children among EAG states by background characteristics in India, NFHS, 2005-06.

Background characteristics	Anemia status by haemoglobin levels				Sample size
	Severe	Moderate	Mild	Not anaemic	
Age groups of children (months)					
6–11	2.30	52.40	28.30	17.00	1696
12–17	5.20	57.60	22.80	14.40	1725
18–23	4.60	57.40	23.30	14.80	1841
24–35	3.90	47.20	26.20	22.60	3519
36–59	2.00	30.90	27.90	39.10	7285
Age groups of mother's (years)					
15–19	4.80	51.70	26.60	16.90	816
20–29	2.80	42.80	26.60	27.80	10678
30–39	3.30	41.30	26.10	29.40	4097
40–49	6.10	34.90	28.60	30.40	475
Place of residence					
Urban	4.10	36.70	25.60	33.60	2919
Rural	2.90	44.00	26.70	26.40	13147
Mothers level of education					
No education	3.40	45.90	26.40	24.40	9815
Up to primary level complete	3.20	41.20	27.10	28.60	2854
Up to secondary level complete	2.70	37.60	27.10	32.50	1606
High school and above	1.80	32.00	26.00	40.30	1790
Fathers level of education					
No education	3.50	47.10	26.50	22.90	5123
Up to primary level complete	2.80	43.10	26.80	27.30	2480
Up to secondary level complete	3.30	41.30	26.40	29.00	6761
High school and above	1.60	33.10	25.90	39.40	1487
Household structure					
Nuclear family	3.40	42.60	26.40	27.60	7210
Joint family	2.80	42.50	26.60	28.10	7710
Wealth quintile					
Poorest	2.80	46.90	27.70	22.50	5594
Poor	3.10	44.40	27.00	25.60	3893
Middle	3.90	41.20	25.60	29.40	2826
Rich	3.00	39.10	25.30	32.60	2188
Richest	2.80	30.90	24.50	41.80	1566
Media exposure					
No	3.10	45.40	27.20	24.30	6378
Yes	3.10	40.90	26.10	29.90	9675
Birth order of the child					
1	2.60	39.30	26.30	31.70	3942
2-3	2.90	42.60	26.70	27.90	6601
4-5	3.40	45.60	26.30	24.80	3451
6+	4.40	44.50	26.80	24.20	2072
Mothers anaemia status					
Severe	12.90	47.70	22.10	17.30	265
Moderate	5.80	52.20	23.40	18.60	2820
Mild	2.80	45.20	27.50	24.50	6607
Not anaemic	1.80	35.50	27.00	35.70	6248

Total	3.58	42.91	26.21	27.31	16065

**Table 3:** Multinomial regression analysis results on anemia status among preschool (6–59 months) children by background characteristics, EAG states in India, NFHS, 2005-06.

Background characteristics	Anaemia status by haemoglobin levels								
	Severe anaemia			Moderate anaemia			Mild anaemia		
	Relative risk (RR)	Confidence interval		Relative risk (RR)	Confidence interval		Relative risk (RR)	Confidence Interval	
		Upper limit	Lower limit		Upper limit	Lower limit		Upper limit	Lower Limit
Age groups of children (months)									
6–11	2.42***	1.53	3.837	4.25***	3.607	5.008	2.45***	2.059	2.92
12–17	7.98***	5.693	11.211	5.41***	4.583	6.401	2.47***	2.061	2.97
18–23	7.33***	5.259	10.239	5.14***	4.392	6.037	2.20***	1.845	2.622
24–35	4.04***	3.092	5.298	2.77***	2.479	3.109	1.67***	1.485	1.891
36–59	1	—	—	1	—	—	1	—	—
Age groups of mothers (years)									
15–19	1.428	0.671	3.036	1.99***	1.356	2.936	1.353	0.903	2.026
20–29	0.693	0.396	1.211	1.50***	1.128	2.018	1.102	0.821	1.478
30–39	0.661	0.393	1.112	1.317*	0.998	1.738	0.982	0.743	1.297
40–49	1	—	—	1	—	—	1	—	—
Place of residence									
Urban	1.319*	0.996	1.746	0.892	0.789	1.009	1.015	0.892	1.155
Rural	1	—	—	1	—	—	1	—	—
Mothers level of education									
No education	1.708**	1.046	2.79	1.40***	1.177	1.675	1.057	0.88	1.271
Primary level complete	1.607*	0.974	2.649	1.23**	1.029	1.471	1.049	0.872	1.263
Secondary level complete	1.4	0.831	2.359	1.149	0.954	1.382	1.064	0.879	1.288
High school and above	1	—	—	1	—	—	1	—	—
Household structure									
Nuclear family	1.011	0.808	1.265	0.928	0.845	1.019	0.98**	0.811	0.988
Joint family	1	—	—	1	—	—	1	—	—
Wealth quintile									
Poorest	1.484	0.877	2.509	1.91***	1.535	2.384	1.90***	1.509	2.394
Poor	1.628*	0.994	2.666	1.64***	1.339	2.024	1.58***	1.279	1.973
Middle	1.637**	1.04	2.577	1.41***	1.17	1.719	1.34***	1.096	1.64
Rich	1.121	0.728	1.726	1.29***	1.086	1.532	1.17*	0.985	1.41
Richest	1	—	—	1	—	—	1	—	—
Media exposure									
No	0.767**	0.596	0.988	0.927	0.832	1.034	0.962	0.857	1.08
Yes	1	—	—	1	—	—	1	—	—
Birth order of the child									
1	0.927	0.472	1.819	0.882	0.663	1.173	0.904	0.668	1.223
2-3	1.14	0.647	2.009	1.012	0.789	1.297	0.98	0.753	1.275
4-5	0.97	0.652	1.442	1.06	0.882	1.273	1.001	0.823	1.217
6+	1	—	—	1	—	—	1	—	—
Mothers anaemia status									
Severe	15.97***	9.533	26.766	2.94***	2.035	4.264	1.61**	1.064	2.447
Moderate	6.271***	4.71	8.351	2.88***	2.532	3.297	1.66***	1.439	1.919
Mild	2.374***	1.821	3.095	1.78***	1.626	1.97	1.44***	1.31	1.601
Not anaemic	1	—	—	1	—	—	1	—	—
States									
Uttaranchal	0.601***	0.347	1.041	0.623***	0.504	0.769	0.926	0.75	1.145
Rajasthan	1.735*	1.221	2.464	0.923	0.776	1.099	0.82**	0.684	0.995
Uttar Pradesh	1.099	0.802	1.506	1.167*	1.018	1.338	1.018	0.881	1.177
Bihar	0.543*	0.343	0.862	1.155	0.971	1.375	1.157	0.964	1.389
Jharkhand	0.322***	0.184	0.565	0.661***	0.547	0.799	0.81**	0.667	0.985
Orissa	0.319***	0.187	0.545	0.564***	0.47	0.676	0.82**	0.689	0.996
Chhattisgarh	0.573***	0.347	0.946	1.065	0.887	1.28	0.861	0.705	1.051
Madhya Pradesh	1	—	—	1	—	—	1	—	—

***P < 0.001, **P < 0.01, *P < 0.05, reference is last category of each variable and not anemic.

## References

[1] Bharati S, Pal M, Chakrabarty S, Bharati P (2013). Socioeconomic determinants of iron-deficiency anemia among children aged 6 to 59 months in India. *Asia-Pacific Journal of Public Health*.

[2] Pasricha S, Black J, Muthayya S (2010). Determinants of anemia among young children in rural India. *Pediatrics*.

[3] Benoist B, McLean E, Egli I, Cogswell M (2008). *Worldwide Prevalence of Anaemia 1993–2005: WHO Global Database on Anaemia*.

[4] International Institute for Population Sciences and Macro International (IIPS and Macro Int.) (2007). *National Family Health Survey (NFHS-3), 2005-06, Key Findings*.

[5] Walter T, de Andraca I, Chadud P, Perales CG (1989). Iron deficiency anemia: adverse effects on infant psychomotor development. *Pediatrics*.

[6] Arlappa N, Balakrishna N, Laxmaiah A, Brahmam GNV (2012). Prevalence of anaemia among rural pre-school children of Maharashtra, India. *Indian Journal of Community Health*.

[7] Gao W, Yan H, Dang S, Pei L (2013). Severity of anemia among children under 36 months old in Rural Western China. *PLoS ONE*.

[8] Jain NB, Laden F, Guller U, Shankar A, Kasani S, Garshick E (2005). Relation between blood lead levels and childhood anemia in India. *American Journal of Epidemiology*.

[9] Brabin BJ, Premji Z, Verhoeff F (2001). An analysis of anemia and child mortality. *Journal of Nutrition*.

[10] Dey S, Gosawmi S, Dey T (2013). Identifying predictors of childhood anaemia in north-east India. *Journal of Health and Population Nutrition*.

[11] Kotecha PV (2011). Nutritional anemia in young children with focus on Asia and India. *Indian Journal of Community Medicine*.

[12] Bentley ME, Griffiths PL (2003). The burden of anemia among women in India. *European Journal of Clinical Nutrition*.

[13] Zhao A, Zhang Y, Peng Y (2012). Prevalence of anemia and its risk factors among children 6–36 months old in Burma. *The American Journal of Tropical Medicine and Hygiene*.

[14] Kapur D, Agarwal KN, Agarwal DK (2002). Nutritional anemia and its control. *Indian Journal of Pediatrics*.

[15] Katzman R, Novack A, Pearson H (1972). Nutritional anemia in an inner-city community. Relationship to age and ethnic group. *The Journal of the American Medical Association*.

[16] Pablo VS, Windom R, Pearson HA (1985). Disappearance of iron deficiency anemia in a high risk infant population given supplemental iron. *The New England Journal of Medicine*.

[17] Behrman RE, Kleigman RM (1994). *Nelson Essentials of Pediatrics: Hematology*.

[18] Oski FA (1993). Iron deficiency in infancy and childhood. *The New England Journal of Medicine*.

[19] Sargent JD, Stukel TA, Dalton MA, Freeman JL, Brown MJ (1996). Iron deficiency in Massachusetts communities: socioeconomic and demographic risk factors among children. *American Journal of Public Health*.

[20] Singh RK (2013). Lifestyle behavior affecting prevalence of anemia among women in EAG states, India. *Journal of Public Health*.

[21] Winichagoon P (2002). Prevention and control of anemia: Thailand experiences. *Journal of Nutrition*.

[22] DeMaeyer E, Adiels-Tegman M (1985). The prevalence of anaemia in the world. *World Health Statistics Quarterly*.

[23] Stoltzfus R, Mullany L, Black RE, Ezzati M, Lopez A, Rodgers A, Murray CJL (2004). Iron deficiency anaemia. *Comparative Quantification of Health Risks: Global and Regional Burden of Disease Attributable to Selected Major Risk Factors*.

[24] Nguyen PH, Nguyen KG, Le MB (2006). Risk factors for anemia in Vietnam. *Southeast Asian Journal of Tropical Medicine and Public Health*.

[25] Ahmed F (2000). Anaemia in Bangladesh: a review of prevalence and aetiology. *Public Health Nutrition*.

[26] Singh MB, Fotedar R, Lakshminarayana J (2009). Micronutrient deficiency status among women of desert areas of western Rajasthan, India. *Public Health Nutrition*.

[27] Singh MB, Fotedar R, Lakshminarayana J, Anand PK (2006). Studies on the nutritional status of children aged 0–5 years in a drought-affected desert area of Western Rajasthan, India. *Public Health Nutrition*.

[28] Konstantyner T, Taddei JAAC, Oliveira MN, Palma D, Colugnati FAB (2009). Isolated and combined risks for anemia in children attending the nurseries of daycare centers. *Jornal de Pediatria*.

[29] Tympa-Psirropoulou E, Vagenas C, Dafni O, Matala A, Skopouli F (2008). Environmental risk factors for iron deficiency anemia in children 12–24 months old in the area of Thessalia in Greece. *Hippokratia*.

[30] Kapil U, Chaturvedi S, Nayar D (1992). National nutrition supplementation programmes. *Indian Pediatrics*.

[31] Vijayaraghavan K, Brahmam GNV, Nair KM, Akbar D, Pralhad Rao N (1990). Evaluation of national nutritional anemia prophylaxis programme. *The Indian Journal of Pediatrics*.

[32] Kandpal E An Evaluation of the Indian Child Nutrition and Development Program.

[33] Gragnolati M, Shekar M, Gupta MD, Bredenkamp C, Lee YK (2005). India’s undernourished children: a call for reform and action. *Health, Nutrition and Population*.

[34] Kapil US (2002). Technical consultation on ‘Strategies for Prevention and Control of Iron-deficiency anemia amongst under three Children in India’. *Indian Pediatrics*.

[35] Chakrabarty A Bihar Children are Still Anemic. http://zeenews.india.com/exclusive/bihar-children-are-still-anemic_5252.html.

[36] Noronha JA, Khasawneh EA, Seshan V, Ramasubramaniam S, Raman S (2012). Anemia in pregnancy-consequences and challenges: a review of literature. *Journal of South Asian Federation of Obstetrics and Gynecology*.

